# Complete Genome Sequence of the Carboxydotrophic Knallgas Bacterium Pseudomonas carboxydohydrogena Strain DSM 1083

**DOI:** 10.1128/mra.01277-22

**Published:** 2023-03-29

**Authors:** Daniel Siebert, Tobias Busche, Ezgi Saydam, Jörn Kalinowski, Christian Rückert-Reed, Bastian Blombach

**Affiliations:** a Microbial Biotechnology, Campus Straubing for Biotechnology and Sustainability, Technical University of Munich, Straubing, Germany; b SynBiofoundry@TUM, Technical University of Munich, Straubing, Germany; c Center for Biotechnology (CeBiTec), Bielefeld University, Bielefeld, Germany; d Medical School East Westphalia-Lippe, Bielefeld University, Bielefeld, Germany; University of Southern California

## Abstract

Pseudomonas carboxydohydrogena is a lithoautotrophic and obligate aerobic alphaproteobacterium, which has the unique ability to utilize CO, CO_2_, H_2_, and mixtures thereof as sole carbon and energy sources. Here, we report the complete genome sequence of type strain DSM 1083 and its close relation to Afipia carboxidovorans strain OM5.

## ANNOUNCEMENT

Strain DSM 1083 was isolated as Seliberia carboxydohydrogena strain Z-1062 from a sewage treatment plant in Russia (1) and renamed Pseudomonas carboxydohydrogena (2). It is a Gram-negative rod-shaped bacterium with a single subpolar flagellum and is able to utilize either CO, CO_2_, and H_2_ or different organic acids for aerobic autotrophic and heterotrophic growth, respectively (2–4).

All subsequently listed programs were run with default parameters unless specified otherwise. The genomic DNA of heterotrophically grown (10 mL tryptone beef extract [TB] medium in 100-mL baffled flasks at 30°C and 120 rpm for 2 days [cf. reference 5]) *P. carboxydohydrogena* strain DSM 1083 (provided by DSMZ GmbH, Germany) was isolated with the NucleoSpin microbial DNA kit (MN GmbH & Co. KG). The same DNA preparation was used to create both sequencing libraries, and no size selection was performed prior to preparation of the Oxford Nanopore Technologies (ONT) library. Long and short DNA reads were generated by Nanopore and Illumina sequencing, respectively. For library preparation, a TruSeq DNA PCR-free high-throughput library prep kit (Illumina) and the SQK-LSK109 ligation sequencing kit (Oxford Nanopore Technologies [ONT]) were used. To generate the short reads, a 2- by 300-nucleotide (nt) run (MiSeq reagent kit v3, 600 cycles) was executed. The long reads were generated on a GridION platform using an R9.4.1 flow cell. Base calling and demultiplexing were performed using Guppy v5.0.16 (6) with the superaccurate base calling model excluding reads shorter than 1,000 nt. Assemblies were done using Flye v.2.9 (7) with “--nano-hq” for the Nanopore long-read data (115,669 reads; read *N*_50_, 14,602 nt) and Newbler v2.8 (8) with options “-large -siom 16 -m -consed -vs phiX.fna” for the Illumina short-read data (5,719,306 reads), resulting in 1 contig (Flye) and 15 contigs in one scaffold (Newbler), respectively. After polishing of the Flye-based assembly using Medaka v1.5.0 (https://github.com/nanoporetech/medaka) with “-m r941_min_sup_g507” and Pilon v1.22 (9) with “–fix-all --changes --frags <BAM>” using Bowtie 2 v2.4.1 (10) with “--no-unal -X 750” for mapping, the two assemblies were combined in Consed v28.0 (11) by creating artificial chromatogram and Phred files for the Flye-assembled contig with BioPerl and adding them to the Consed project created by Newbler. No trimming of contigs was performed as Flye automatically trims circular contigs. Manual finishing was done by comparing all Newbler contigs to the single contig from Flye, resolving all differences based on visual inspection of the read data at the respective positions and ensuring that the Flye-generated contig was continuously covered by contigs produced by Newbler. The resulting single contig representing the circular genome with 426.6-fold sequence coverage was annotated using the PGAP pipeline v2022-10-03.build6384 (12, 13). After the initial annotation, the sequence was rotated to start at the position of *dnaA* using Seqret v6.6.0.0 (14) and annotated a second time. With a size of 3,271,440 bp and a G+C content of 62.45%, the genome contains a total of 3,160 genes made up of 3,076 intact coding sequences (CDS), 1 rRNA gene operon of 3 rRNAs, 49 tRNAs, 3 noncoding RNAs (ncRNAs), and 29 pseudogenes.

**FIG 1:**
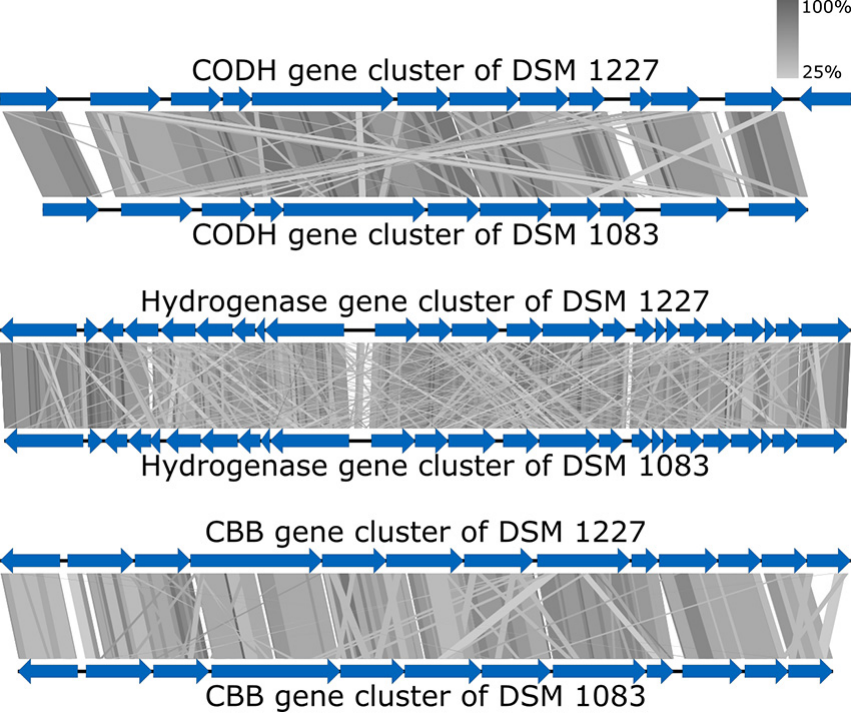
Alignment of the gene clusters coding for CO dehydrogenases (CODH) (from *coxB* to *coxK* in DSM 1227), hydrogenases (from OCA_pHCG300760 to *hoxA* in DSM 1227), and Calvin-Benson-Bassham (CBB) cycle enzymes (from *cbbR* to OCA5_pHCG300520 in DSM 1227) of *P. carboxydohydrogena* DSM 1083 and *A. carboxidovorans* DSM 1227 (16) via tBLASTx (15) and Easyfig 2.2.5 (17). Blue arrows represent single genes of gene clusters. Similarities are shown by gray gradients.

The genome sequence presented here indicates a close relationship of Afipia carboxidovorans and *P. carboxydohydrogena*, represented by high sequence similarities in the bacterial chromosome via BLASTn (15) (query coverage, 78%; percent identities, 83.55%) and even for all relevant genes necessary for autotrophic growth (coverage of ≥88%, identity of ≥90%) (Fig. 1).

### Data availability.

All sequencing data as well as the annotated genome can be accessed via BioProject PRJNA901395. SRA read data are available via SRX18291734 (ONT) and SRX18291735 (Illumina), and the annotated genome sequence can be retrieved via the accession no. CP113162.

## References

[1] Zavarzin GA, Nozhevnikova AN. 1977. Aerobic carboxydobacteria. Microb Ecol 3:305–326. doi:10.1007/BF02010738. 24233667

[2] Meyer O, Lalucat J, Schlegel HG. 1980. *Pseudomonas carboxydohydrogena* (Sanjieva and Zavarzin) comb. nov., a monotrichous, nonbudding, strictly aerobic, carbon monoxide-utilizing hydrogen bacterium previously assigned to Seliberia. Int J Syst Bacteriol 30:189–195. doi:10.1099/00207713-30-1-189.

[3] Cypionka H, Meyer O, Schlegel HG. 1980. Physiological characteristics of various species of strains of carboxydobacteria. Arch Microbiol 127:301–307. doi:10.1007/BF00427208.

[4] Meyer O, Schlegel HG. 1983. Biology of aerobic carbon monoxide-oxidizing bacteria. Annu Rev Microbiol 37:277–310. doi:10.1146/annurev.mi.37.100183.001425. 6416144

[5] Siebert D, Busche T, Metz AY, Smaili M, Queck BAW, Kalinowski J, Eikmanns BJ. 2020. Genetic engineering of *Oligotropha carboxidovorans* strain OM5-a promising candidate for the aerobic utilization of synthesis gas. ACS Synth Biol 9:1426–1440. doi:10.1021/acssynbio.0c00098. 32379961

[6] Perešíni P, Boža V, Brejová B, Vinař T. 2021. Nanopore base calling on the edge. Bioinformatics 37:4661–4667. doi:10.1093/bioinformatics/btab528. 34314502 PMC8665737

[7] Kolmogorov M, Yuan J, Lin Y, Pevzner PA. 2019. Assembly of long, error-prone reads using repeat graphs. Nat Biotechnol 37:540–546. doi:10.1038/s41587-019-0072-8. 30936562

[8] Miller JR, Koren S, Sutton G. 2010. Assembly algorithms for next-generation sequencing data. Genomics 95:315–327. doi:10.1016/j.ygeno.2010.03.001. 20211242 PMC2874646

[9] Walker BJ, Abeel T, Shea T, Priest M, Abouelliel A, Sakthikumar S, Cuomo CA, Zeng Q, Wortman J, Young SK, Earl AM. 2014. Pilon: an integrated tool for comprehensive microbial variant detection and genome assembly improvement. PLoS One 9:e112963. doi:10.1371/journal.pone.0112963. 25409509 PMC4237348

[10] Langmead B, Salzberg SL. 2012. Fast gapped-read alignment with Bowtie 2. Nat Methods 9:357–359. doi:10.1038/nmeth.1923. 22388286 PMC3322381

[11] Gordon D, Abajian C, Green P. 1998. Consed: a graphical tool for sequence finishing. Genome Res 8:195–202. doi:10.1101/gr.8.3.195. 9521923

[12] Li W, O’Neill KR, Haft DH, DiCuccio M, Chetvernin V, Badretdin A, Coulouris G, Chitsaz F, Derbyshire MK, Durkin AS, Gonzales NR, Gwadz M, Lanczycki CJ, Song JS, Thanki N, Wang J, Yamashita RA, Yang M, Zheng C, Marchler-Bauer A, Thibaud-Nissen F. 2021. RefSeq: expanding the Prokaryotic Genome Annotation Pipeline reach with protein family model curation. Nucleic Acids Res 49:D1020–D1028. doi:10.1093/nar/gkaa1105. 33270901 PMC7779008

[13] Tatusova T, DiCuccio M, Badretdin A, Chetvernin V, Nawrocki EP, Zaslavsky L, Lomsadze A, Pruitt KD, Borodovsky M, Ostell J. 2016. NCBI prokaryotic genome annotation pipeline. Nucleic Acids Res 44:6614–6624. doi:10.1093/nar/gkw569. 27342282 PMC5001611

[14] Madeira F, Pearce M, Tivey ARN, Basutkar P, Lee J, Edbali O, Madhusoodanan N, Kolesnikov A, Lopez R. 2022. Search and sequence analysis tools services from EMBL-EBI in 2022. Nucleic Acids Res 50:W276–W279. doi:10.1093/nar/gkac240. 35412617 PMC9252731

[15] Altschul SF, Madden TL, Schäffer AA, Zhang J, Zhang Z, Miller W, Lipman DJ. 1997. Gapped BLAST and PSI-BLAST: a new generation of protein database search programs. Nucleic Acids Res 25:3389–3402. doi:10.1093/nar/25.17.3389. 9254694 PMC146917

[16] Volland S, Rachinger M, Strittmatter A, Daniel R, Gottschalk G, Meyer O. 2011. Complete genome sequences of the chemolithoautotrophic *Oligotropha carboxidovorans* strains OM4 and OM5. J Bacteriol 193:5043. doi:10.1128/JB.05619-11. 21742883 PMC3165685

[17] Sullivan MJ, Petty NK, Beatson SA. 2011. Easyfig: a genome comparison visualizer. Bioinformatics 27:1009–1010. doi:10.1093/bioinformatics/btr039. 21278367 PMC3065679

